# Estimating the economic incentives necessary for eliminating child labor in Ghanaian cocoa production

**DOI:** 10.1371/journal.pone.0217230

**Published:** 2019-06-07

**Authors:** Jeff Luckstead, Francis Tsiboe, Lawton L. Nalley

**Affiliations:** 1 Department of Agricultural Economics, University of Arkansas, Fayetteville, Arkansas, United States of America; 2 Department of Agricultural Economics, Kansas State University, Manhattan, Kansas, United States of America; International Institute of Tropical Agriculture (IITA), MALAWI

## Abstract

Concerns about the use of child labor in West African cocoa production became widespread in the early 2000s in many high-income countries. In 2015 in Ghana, 91.8% (or a total of 878,595) of the children working in the cocoa sector were involved in a form of hazardous work. Child labor in cocoa production is not just a symptom of poverty but also a contributing factor, as children often forgo a formal education to work in cocoa orchards. Current Ghanaian law prohibits child labor, but, with many cocoa households living in poverty, child labor becomes a necessity for survival, and as such, current child labor laws are rarely enforced. Therefore, an effective policy that eliminates child labor could compensate farmers by providing an economic incentive. In this paper, we develop and calibrate a farm household model to estimate the cocoa price premium necessary to eliminate child labor from cocoa production while leaving the farm household welfare unchanged. This welfare-neutral price premium removes the negative effects of eliminating child labor for the farm household. Varying degrees of child labor exists, with certain forms posing a greater risk to children’s wellbeing. The results show that eliminating the worst forms of child labor would require a cocoa price premium of 2.81% and eliminating regular work (non-hazardous work but over the maximum hours allowed for a child) and the worst forms would require an 11.81% premium, which could be paid for by the well-established Ghanaian Cocoa Marketing Board. An incentive for the Cocoa Marketing Board to pay the price premium and monitor and enforce this policy would be the ability to differentiate their cocoa as child-labor free and not lose market share to countries who cannot currently certify this practice.

## Introduction

In the early 2000s, concerns about child labor use in cocoa production became widespread in the United Kingdom and other high-income countries following newspaper and documentary allegations of the use of child slaves in West Africa [1, 2, 3, 4]. The allegations, which focused on the discovery of enslaved young men working on Ivorian cocoa farms, later spread concerns about such practices being used in other West African cocoa producing countries. The cocoa industry promptly reassessed its influence over the social responsibility regarding human rights and welfare in the cocoa supply chain. Part of the cocoa industries’ concern, outside of the human rights arena, is that U.S. Executive Order 13126 prohibits federal agencies from purchasing goods made using child labor.

While executive orders in the United States and consumer demand in the European Union expressed vocal concern, these responses were merely reactions and not solutions to child labor. The most common response to child labor throughout high-income countries is to simply legislate against its usage. However, much of Sub-Saharan African agriculture is conducted by peasant farmers and is much harder to regulate; thus, legislation is often ineffective. Additionally, according to [5], the involvement of children in cocoa production is an age-old tradition of imparting cocoa farming skills to the younger generation to take over family farms. The tradition of passing farm skills through the generations adds additional difficulties in legislating against child labor.

Almost one in three children between the ages of five and fourteen are economically active in Africa, compared with fewer than one in five in Asia and one in six in Latin America [6]. According to [7], when child labor occurs in mass (as in the case of the West African cocoa industry), it is likely a symptom of poverty. This is because, when poverty is widespread, parents are compelled to send children to work for survival reasons, reducing the time children spend in school. In addition to being a symptom, child labor is also a contributing factor to poverty, making it increasingly difficult to achieve economic independence, as households rely on child work for support. For households with higher income, education is a necessity, not a luxury good, and adults can internalize the value of child education [8, 9, 10].

In 2001, the Harkin-Engle protocol was signed in the United States, supporting the efforts to end the “worst forms” of child labor in West African cocoa production by providing aid in the amount of ten million dollars [11]. As part of this effort, Tulane University conducted a compressive study in Ghana and Cote d’Ivoire––the two largest cocoa producers globally. The results showed that the number of children aged 5–17 years working in cocoa production, participating in child labor in cocoa production, and doing hazardous work in cocoa production actually grew by 24%, 21%, and 18%, respectively, between the 2008/09 and 2013/14 cocoa growing seasons [12]. In Ghana, there were reductions, albeit small, in the percentage of children working in cocoa production classified as hazardous work [12].

Since the signing of the Harkin-Engle Protocol, the number of children working in hazardous cocoa production across West Africa has increased partially due to the introduction of high-yielding and/or disease-resistant cocoa varieties that require more labor to harvest and process [12]. To meet the Harkin-Engle protocol challenge of a 70% reduction of the worst forms of child labor by 2020, 1.5 million children in both Ghana and Cote d’Ivoire will have to be removed from hazardous work. In June of 2016, the U.S. Department of Labor hosted the Child Labor in Cocoa Coordinating Group (CLCCG) to discuss progress and the challenges of the Harkin-Engel Protocol. During the meeting, Tim McCoy, the President of the World Cocoa Foundation (an NGO that represents governments of cocoa producing regions, cocoa buyers, and cocoa processors), highlighted the need for public-private partnerships to combat and prevent child labor. Further, he emphasized that the cocoa community needed better supply chain-based child labor monitoring and remediation [13]. Thus, it would appear that governments and other public entities cannot eliminate child labor alone in cocoa producing countries and need assistance from private industry. This private assistance could come in the unlikely form of the outright ban on purchasing cocoa from countries that violate child labor laws or the more likely form of labeling and paying premiums for those countries who are mitigating child labor in cocoa production.

As activists, consumers, and politicians continue to be restless about reducing child labor in West African cocoa production, the possibility of labeling “child labor free cocoa” becomes a real possibility [14]. However, without tangible economic incentives, cocoa producers in Ghana and Cote d’Ivoire may not have the time or capacity to respond. This could lead to increased demand from consumers in high-income countries for cocoa from other producing regions globally that have reduced or no child labor issues. This scenario may simply exacerbate the poverty issue in Ghana and Cote d’Ivoire, which in turn could lead to increased, not decreased, levels of child labor in these poverty-stricken regions. However, if an economic incentive like a premium that would pay for cocoa produced in these countries with no child labor existed, then a tangible reduction in child labor may be experienced.

The highest rates of child labor in Africa (> 40%) are found in Mali, Burkina Faso, Niger, Kenya, Uganda, Burundi, and Rwanda—none of which are major cocoa producers [6]. Most of these countries rely on non-cash crops, which are not exported. That is, most of the agriculture in the countries with the highest rates of child labor use this labor for producing food in a subsistence farming framework. In fact, one estimate found that less than 3% of child laborers work in export-oriented agriculture [15]. This raises an interesting question: Do consumers in high-income countries want to end all child labor or only the child labor that is a function of products they consume? Moreover, if consumers in high-income countries demand child-free labor to produce cash crops for exportation like cocoa, would that child labor simply reappear in subsistence farming of other crops such as maize, sorghum, and rice that would be consumed domestically?

Given that legislation of informal farming in Sub-Saharan Africa has proven impractical from an enforcement sense, this paper contributes to the existing literature on child labor in cocoa production by calculating the necessary economic incentive in the form of a fair-trade price premium to entice cocoa producers in Ghana to eliminate hazardous child labor. Given the empirical fact that cocoa production in Ghana is predominantly done by small-scale household farms [16, 17, 18, 19], calculating the price premium to eliminate hazardous child labor calls for a modeling of the household itself. A Farm Household Model (FHM) analyzes factors that influence simultaneous production and consumption (of both cocoa and food staples) decisions of farmers, demand for production inputs (fertilizer, fungicide, capital, and land), and labor/leisure decisions in a theoretically consistent fashion. As such, simulation analysis can be used to illustrate the relative outcomes of policies [20].

The objectives of this study are to (1) formulate and calibrate an FHM for cocoa producers in Ghana who participate in child labor; (2) quantify the effects of eliminating child labor on the equilibrium price, production, and welfare in the cocoa and domestic food markets; and (3) calculate the price premium that would be necessary to make a cocoa-growing household indifferent to the elimination of child labor.

A theoretical model [21] was developed to analyze the possibility of using international transfers to buy-out child labor. The model quantifies the Pareto optimal transfers and shows that the elimination of child labor is possible. They conclude that the transfers needed to eradicate child labor immediately significantly exceed the willingness to pay by consumers in more developed countries. In this study, using Ghana as a case study, an empirical model is used to calculate what the money transfer ($/kg of cocoa) would need to be to eliminate child labor while leaving cocoa farmers indifferent. If the estimated premium is relatively small enough, then activists and consumers could incentivize the reduction of child labor via price premiums paid to producers, instead of relying on political enforcement that has historically been less effective given the informal nature of the cocoa market.

## Literature review

### Child labor

The International Labor Organization (ILO) classifies child labor into three categories: i) worst forms, ii) regular work and worst forms, and iii) light and regular work and the worst forms. The worst forms of child labor are bisected into two categories. The first, hazardous work, is defined as “employment in industries and occupations designated as hazardous or work for long hours and/or at night in industries and occupations not designated as hazardous.” The second, *worst forms other than hazardous work*, is defined as “children trafficked for work; forced and bonded child labor; commercial sexual exploitation of children; use of children for illicit activities and armed conflict.” While there are many children throughout Africa involved in the *worst forms other than hazardous work*, this paper and the cocoa industry is focusing on the more prevalent form of child labor in the cocoa industry: *hazardous work*. Regular work and worst forms constitute employment below the general minimum working age in addition to the worst forms of child labor. Light and regular work and the worst forms also include, in addition to regular work and the worst forms, employment below the minimum working age for light work. According to the ILO, light work includes activities that are not harmful to a child’s health or development and does not affect their school attendance, other vocational training, etc. Table 1 highlights what the International Labor Organization classifies as child labor by age and work type/duration.

**Table 1:** International labor organization’s classification of child labor types, ages, and focus areas.

	Non-Hazardous		Hazardous
Age	Light Work	Regular Work	Hazardous Work
	Non-Hazardous in nature and less than 14 hours/week	Non-Hazardous in nature and between 14 and 42 hours/week	Hazardous in nature OR Non-Hazardous for more than 42 hours/week
Under 12	Needs eliminating	Needs eliminating	Needs eliminating
12 to 14		Needs eliminating	Needs eliminating
14 to 17			Needs eliminating

Source: [6]

Most theoretical treatments of entry into *worst forms* assume that children are more likely to enter *worst forms* when their alternative employment opportunities are limited and the economic return is large. In Nepal, paternal disability was found to be a strong predictor of entry of children into the worst forms, and the presence of productive assets reduces the child’s risk of engaging in the worst forms [22]. The study [22] finds that that debt bondage is an important driver in child labor in agrarian communities in low-income countries. Families in debt often turn to child labor to ensure the loan is paid in full. [23] modeled the effects of a policy banning child labor. They found that when a ban is used as the sole instrument to eradicate the worst forms of child labor, policymakers run the risk of mixing willful child labor and child labor resulting from enslavement or deception. In the case of child labor in the mining industry in Northern Ghana, [24] found the majority of children engaged in mine-related work appears interested in attending school, and many continue to engage in arduous mining activities in order to pay for their school fees. A rural household model was implemented and econometric analysis shows that, on average, market imperfections (e.g., poorly defined land rights, labor and credit market information asymmetries, and lack of enforcement) lead to enhanced levels of child labor; however, households with large landholdings supply more child labor while medium landholdings supply less when market imperfections arise [25]. An inverse relationship was estimated between child labor and per capita landholding [26]. However, families with mid-range landholdings tend to use more child labor, as the marginal value of farm work rises faster than education.

There are examples of agricultural policy reforms reducing child labor and increasing the educational attainment of children globally. In 1993, more than 50% of the children in Vietnam worked full time. In 1998, to increase its rice exports and reduce child labor, Vietnam lifted its restrictions on rice exports. Consequently, the domestic price of rice rose, which afforded parents the ability to send their children to school and reduced the share of 6–15-year-olds working at least seven hours a day from 57% to 38% [27].

In Côte d’Ivoire, Nestlé introduced the Child Labor Monitoring and Remediation System (CLMRS), a program to address child labor and support children of farmers and workers [28]. The supply chain of cocoa production is one of the largest impediments to eliminating labor in the cocoa sector. Unlike other commodities grown in Africa, cocoa is grown in remote areas and can take weeks (or months) to come to market. So, by its very biological nature, it is more difficult to determine if cocoa (which is often sold and blended between farms many times before sold to a wholesaler) has been produced with child labor. Thus, historically, child labor in cocoa has been less regulated, less enforced, and more prevalent than in crops with a shorter supply chain.

### Child labor in Ghanaian cocoa production

Ghanaian law states children under 18 are prohibited from working on a farm for more than “three hours per day or more than 18 hours per week (for children on weekends, holidays, and/or children who have completed school)” or “more than 2 hours/day on a school day” [29]. The child labor laws in Ghana provide a framework specifying that “going to or returning from the farm alone” and “working on farm between the hours of 6.00 p.m. and 6.00 a.m.” are prohibited for children. In addition, a child cannot be “withdrawn from school during cocoa season to do farm work” and cannot work “full time on farm [while] not attending formal/non-formal school (applicable to children under 15 years).” Section 91 stipulates that hazardous employment is proscribed for all children under 18 years of age, and Section 87 forbids the engagement of a child in “exploitative child labor” that “deprives the child of its health, education or development.” Any of these activities are considered *hazardous work* under Ghana’s framework [29]. Furthermore, a Ghanaian law specific to cocoa states the following activities to be *hazardous child labor activities*: clearing of forest or felling trees, working with agrochemicals, breaking cocoa pods with a breaking knife, climbing trees higher than three meters, and harvesting overhead cocoa pods with a harvesting hook. However, despite legislation, these activities are still undertaken by children in Ghanaian cocoa production with little disincentive to cease.

A Tulane study [30] found that the total number of children (5–17 years old) working in the cocoa sector in the previous twelve months was 997,375, which accounts for 46.2% of all the children in the cocoa-growing areas of Ghana for the 2008/09 growing season. Ninety-five percent of the children working in the cocoa sector—equivalent to 43.9% of all children in the area—were involved in excessive child labor. The report also found that 93% of the children working in the cocoa sector were also involved in hazardous work. In a follow-up survey, [12] found that there were 957,398 children working in the Ghanaian cocoa industry in the 2013/14 cocoa growing season, 98.1% of which were involved with hazardous work. Hazardous work involved both sexes, with 60% (507,820) of all males and 40% (370,774) of all females involved in cocoa production undertaking work classified as *hazardous work*. The report also found that 39.5% of the 878,595 children found to be conducting hazardous work were between the ages of 5–11 years old. The report found that, when classifying those children who were found to be conducting hazardous work in the 2013/14 growing season, 74.6% had to carry heavy loads, 33.1% handled agro-chemicals, and 84.3% handled sharp tools.

When analyzing whether the International Labor Organization’s policy in Ghana of increasing the education rate of children has the potential of eliminating child labor in farm and non-farm households, [31] found that the Ghanaian government should provide adequate remuneration for workers and lobby/bargain for comprehensive prices for agricultural products (especially at the international level). Consequently, households do not need to diversify their income portfolios by moving on-farm to off-farm child labor.

Given the large absolute number and relative percentage of total children that are both involved in cocoa production and who undertake hazardous work, it appears that simple government regulation/education policies and international pressure is currently not sufficient to eliminate hazardous work for children in cocoa production in Ghana. As such, this study explores what economic incentive, in the form of a price premium (similar to the approach put forth by [31]), would be necessary to make a cocoa-growing household indifferent to using child labor. This approach is unique because, unlike mandates and international pressure from activists which punish poverty-stricken households who use hazardous child labor, this study estimates a premium to incentivize its reduction.

### The Ghanaian cocoa marketing board

The role of the Ghanaian Cocoa Marketing Board (COCOBOD) in cocoa production is twofold: to (i) administer the process of internal marketing and (ii) hold the monopsony power in internal cocoa purchasing from farmers and monopoly power in exporting cocoa [32]. In purchasing cocoa, COCOBOD sets the farm gate cocoa price in Ghana as a share of the net free-on-board price. For the period 2010 to 2013, the farm gate price for Ghana was set at 77.81% of the net free-on-board price [33]. This price setting regime enables COCOBOD to have working capital to stabilize the farm gate price and carry out its mandate.

One of the stated goals of the Ghanaian COCOBOD is to “uphold social conscience, treat our farmers fairly and maintain a mutually beneficial relationship with all stakeholders” [34]. In 2012, Mr. Kwabena Asante Poku, Deputy Chief Executive of COCOBOD, was quoted stating that “as the regulator of the cocoa industry, [COCOBOD has] had worked tirelessly with partners and other stakeholders to sensitize cocoa farmers to conduct their operations in a professional manner, and ensure that their children attend school during school hours without any hindrance” [35]. These comments, along with the mission statement of the COCOBOD, implies that COCOBOD has internalized the child labor issue facing their industry and are willing to take proactive measures to remedy it.

## Farm household model: Child labor and cocoa production

This study develops a farm household model [20] that accurately reflects the production and market conditions of the Ghanaian cocoa industry. While the farm household produces cocoa as a cash crop, they also cultivate food crops (such as cassava, yam, and maize) [36, 37], mainly for subsistence consumption. Since cocoa is a cash crop, all production is surplus and sold at the farm gate price. The farm household uses income from cocoa production and other sources to purchase staple foods that are not met by the household’s own food production as well as non-food items. The household structure treats farmers as both semi-commercial and semi-subsistence with elements of both producer and consumer theory. Consequently, farmers make both production versus consumption and work versus leisure decisions simultaneously.

Given that labor is a principal variable input for cocoa production and the involvement of children in cocoa production activities, labor is a key part of the model strategy. Benevolent parents make decisions on their own time allocation and also the time allocation of their children. The cocoa-producing household utilizes its own (adults and children) labor and hired labor in cocoa production due to the financial importance of this crop. By contrast, for food production, the household only utilizes its own (adults and children) labor. Therefore, adults split their time between cocoa production, food production, and leisure. However, in addition to working in cocoa and food production and engaging in leisure, children also attend school, which puts a higher demand on children’s time compared to adults.

This study assumes that children are less productive per hour of work than adults, and (more importantly) hired, adult, and child labor are *imperfect substitutes* because hired labor and children are not able to manage the farm and make production decisions. The imperfect substitution in labor types implies that separability between income and expenditure fails. To analyze the impact of a restriction in child labor, the model includes a constraint on the amount of time children can dedicate to work in cocoa production. Finally, the price of cocoa, food, and wage rate are endogenous as these markets clear, whereas the price for non-labor inputs and non-food goods are exogenous.

A representative cocoa farm household is assumed to maximize its utility (*U*) from food consumption (*F*), non-food consumption (*M*), leisure for adults (*L*_*a*_) and child (*L*_*e*_), and child education (*E*_*e*_). Preferences are represented by an additively separable Stone-Geary utility function:

$$\max_{F,M,L_{i},E_{e}}\left[U\right]=\max_{F,M,L_{i},E_{e}}\left[{\left(F-d\right)}^{\alpha_{F}}M^{\alpha_{M}}L_{a}^{\alpha_{a}}L_{e}^{\alpha_{e}}+{\kappa E}_{e}^{\alpha_{E}}\right],$$ (1)

where *d* is the subsistence level of consumption of staple foods, *α*_*i*_s are consumption share parameters, and *κ* is a scale parameter. The subsistence parameter implies that *d* units of food must be consumed before the household will spend income on *M* or *E*_*e*_. Consequently, as income rises, the portion of income dedicated to food consumption falls, which satisfies Engel’s Law. The first set of terms on the right side of (1) express utility of both consumption goods (*F* and *M*) and leisure (*L*_*a*_ and *L*_*e*_), while the second set of terms represents net present value of future utility of children based on their obtained level of education *E*.

Households produce cocoa (*S*_*c*_) according to the decreasing returns-to-scale Cobb-Douglas production function:

$$S_{c}=Z_{c}{\left(L_{c}\right)}^{\beta_{L}}\prod_{j=1}^{4}{\left(x_{j}\right)}^{\beta_{j}},(\sum \beta <1),$$ (2)

$$L_{c}={\left(\psi_{hc}L_{hc}^{\rho }+\psi_{ac}L_{ac}^{\rho }+\psi_{ec}L_{ec}^{\rho }\right)}^{\frac{1}{\rho }},$$ (3)

where *Z*_*c*_ is the cocoa productivity parameter, *L*_*c*_ is a constant elasticity of substitution (CES) composite of hired (*L*_*hc*_), adult (*L*_*ac*_), and child (*L*_*ec*_) labor employed in cocoa production, *ψ*_*ic*_ are value shares, *ρ* is the CES parameter with elasticity of substitution *σ* = 1/(1−*ρ*), *x*_*j*_ are non-labor inputs of production (*j* = 1 for fertilizer, 2 for insecticide/pesticide, 3 for other agro-chemicals, and 4 for equipment/capital), and *β*_*j*_ are output elasticities of production inputs. Because of the perennial nature of cocoa trees and a life cycle of about 25 years, land is assumed to be a fixed factor of cocoa production and dictates the degree of decreasing returns-to-scale in the production function.

The representative cocoa household also produces food (*S*_*F*_) for household consumption according to the Cobb-Douglas production function:

$$S_{f}=Z_{f}L_{f}^{\delta_{L}}A_{f}^{\delta_{A}},(\sum \delta =1),$$ (4)

$$L_{f}={\left(\psi_{af}L_{af}^{\rho }+\psi_{ef}L_{ef}^{\rho }\right)}^{\frac{1}{\rho }},$$ (5)

where *Z*_*F*_ is the productivity parameter, *L*_*f*_ is a CES composite of adult (*L*_*af*_) and child (*L*_*ef*_) labor used in food production, *ψ*_*if*_ are share parameters, *A*_*f*_ is land use, and δ_*i*_ are output elasticities of production inputs. Households do not utilize hired labor in food production because cultivation typically takes place on small plots and food is only for home consumption.

The cash income constraint is

$$P_{F}F+P_{M}M+P_{E}E_{e}=(1+\sigma )P_{c}S_{c}-wL_{hc}+P_{F}S_{F}-\sum_{j=1}^{4}[P_{j}x_{j}]-rA+T,$$ (6)

where *w* and *r* are the wage rate and rental rate of land, *P*_*j*_ are input prices for the *j*^th^ non-labor input, *P*_*F*_ is the price of food, *P*_*M*_ is the price of non-food, *P*_*E*_ is the price of education, and *P*_*c*_ is the price of cocoa. The parameter *σ* is an *ad valorem* price subsidy used to compensate households for restricting child labor, and *T* is non-cocoa income. Because food production follows constant returns-to-scale technology, net profits are zero and food production does not explicitly contribute to farm income, although it does reduce the net cost of food items purchased from the market.

The total time constraint for adults is

$$t_{a}=L_{ac}+L_{aF}+L_{a},$$ (7)

where total time availability for adults (*t*_*a*_) equals time spent on cocoa production (*L*_*ac*_), food production (*L*_*aF*_), and leisure (*l*_*a*_). The total time constraint for children is

$$t_{e}=L_{ec}+L_{eF}+E_{e}+L_{e},$$ (8)

where total time availability for child (*t*_*e*_) equals time spent on cocoa production (*L*_*ec*_), food production (*L*_*eF*_), formal education (*E*_*e*_), and leisure (*L*_*e*_).

During the 2013/14 growing season, 61.28% of the children working in agriculture who were considered child labor were working in cocoa production, while only 38.72% were working in non-cocoa agricultural production [12]. Furthermore, both domestic (e.g., Ghana Cocoa Board [COCOBOD]) and international (e.g., World Cocoa Foundation) agencies are only concerned with child labor in cocoa production and not all Ghanaian agriculture. Therefore, this study considers a labor restriction only for cocoa production:

$$L_{ec}\leq {\bar{L}}_{e},$$ (9)

where ${\bar{L}}_{e}$ is the maximum amount of time children can spend in cocoa production. This study assumes that, if binding, this restriction limits the time children spend engaged in child labor while leaving acceptable forms of children working unaffected.

The study simplifies the budget constraint (6) and the child labor constraint (9) as follows. Solve for *L*_*hc*_ from Eq (3), *L*_*ac*_ from Eq (7), and *L*_*ec*_ from Eq (8). Then, substitute these solved variables, along with *S*_*c*_ and *S*_*f*_ from Eqs (2) and (4) and *L*_*f*_ from Eq (5), into the budget constraint (6) to obtain

$$P_{F}F+P_{M}M+P_{E}E_{e}=\pi_{c}+\pi_{F}+T,$$ (10)

where the profits from cocoa production (*π*_*c*_) and staple food production (*π*_*F*_) are

$$\pi_{c}=(1+\sigma )P_{c}Z_{c}{\left(L_{c}\right)}^{\beta_{L}}\prod_{j=1}^{4}{\left(x_{j}\right)}^{\beta_{j}}-w\psi_{hc}[L_{c}^{\rho }-\psi_{ac}{\left(t_{a}-L_{aF}-l_{a}\right)}^{\rho }-\psi_{ec}{\left(t_{e}-L_{eF}-E_{e}-l_{e}\right)}^{\rho }]-\sum_{j=1}^{4}\left(P_{j}x_{j}\right),$$

$$\pi_{F}=P_{F}Z_{f}{\left(\psi_{af}L_{af}^{\rho }+\psi_{ef}L_{ef}^{\rho }\right)}^{\frac{\delta_{L}}{\rho }}A_{f}^{\delta_{A}}-rA_{f}.$$

The left side of Eq (10) gives total household expenditures on food, non-food items, and child education. The right side of Eq (10) is income from profits from cocoa and staple food production and non-farm income *T*. Further, substituting *L*_*ec*_ from Eq (8) into the child labor restriction (9) yields

$$t_{e}-L_{ef}-E_{e}-L_{e}\leq {\bar{L}}_{e}.$$ (11)

The budget constraint (10) brings out an important property of this model discussed at the start of this section: the separability property, where consumption and production decisions are made independently, fails to hold. The breakdown of the separability property stems from hired, child, and adult labor being imperfect substitutes. An increase in adult leisure, which implies a decrease in the amount of adult labor used in cocoa production, directly impacts production decisions because the decline in adult labor cannot be met by an equal increase in either hired or child labor. The opposite holds true for a decline in adult leisure. The same logic also follows for changes in child leisure.

Mathematically, the breakdown of the separability property is seen in *π*_*c*_ because *L*_*a*_, *E*_*e*_, *L*_*e*_, *L*_*aF*_, and *L*_*eF*_ cannot be isolated from the expenditure part of the profit equation. Consequently, in the first-order conditions for *L*_*a*_, *L*_*e*_, and *E*_*e*_, marginal utility is equal to a term that is a function of the wage rate and total labor *L*_*c*_ used in cocoa production, among other variables (see the corresponding Karush–Kuhn–Tucker in Appendix A in S1 File). Similarly, in the first-order conditions for *L*_*c*_, the marginal value product of total labor *L*_*c*_ is equal to a term that is a function of the wage rate, leisure (*L*_*a*_ and *L*_*e*_), and education decision (*E*_*e*_) variables. Consequently, a direct link between utility and production decisions exists, which implies that separability does not hold.

Utility (1) is maximized subject to the cash income constraint (10) and the child farm labor constraint (11) to endogenously solve for optimal consumption, leisure, and education (*F*, *M*, *L*_*a*_, *L*_*e*_, and *E*_*e*_), labor and input allocation for cocoa production (*L*_*c*_ and *x*_*j*_ for *j* = 1,2,3,4), and labor and land allocations for food production (*L*_*af*_, *L*_*ef*_, and *A*_*f*_).

The price for cocoa, wage rate, and food price are solved through their respective market clearing conditions. The world cocoa price is endogenous because cocoa farms in Ghana account for about 24% of total world exports [38], the second largest in the world, and the COCOBOD sets the farm gate price of cocoa as a share of the world cocoa price. Also, according to FAOStat, between 1961 and 2013, on average, 95% of Ghanaian cocoa production was exported. One of the objectives of the COCOBOD is to buffer farmers from year-to-year world price shocks. However, since this analysis considers the effects of child labor policy on world cocoa markets, any structural change in the world price will be passed on to the farmers. The market-clearing condition, which determines the world cocoa price, is such that Ghanaian cocoa production equals world demand for Ghanaian cocoa:

$$S_{c}=D_{c}P_{c}^{\eta }.$$ (12)

Supply of Ghanaian cocoa on the left side is given by Eq (2). Demand on the right side is the world demand for Ghanaian cocoa with scale parameter *D*_*c*_>0 and elasticity parameter demand *η*<0. This residual demand function is calculated as total world demand for cocoa minus supply of cocoa by all non-Ghanaian countries, which allows the analysis to maintain focus on the Ghanaian cocoa market.

As child labor is reduced, cocoa-producing households are likely to hire more labor, which could raise the wage for hired work. The labor market-clearing conditions, which determines the wage rate, is such that the supply of agricultural labor supply equals labor demand by Ghanaian cocoa farms:

$$\omega_{l}w^{\gamma_{l}}=L_{hc}.$$ (13)

The right side is supply of hired workers where *ω*_*l*_>0 is a scale parameter and *γ*_*l*_>0 is the elasticity of labor supply. The left side is demand for hired workers defined in Eq (3).

While cocoa farmers produce some food for consumption, it is not sufficient to meet the food requirements of the household. As a result, a portion of the total food consumption is purchased from the food market. A change in child labor practices could result in the cocoa household purchasing more or less food from the market, which could impact the price. The food market-clearing condition is such that total supply of food is equal to demand by cocoa-producing households:

$$S_{f}+\omega_{f}=F.$$ (14)

The first term on the right side is food production by cocoa farmers, given by Eq (4), and the second term is food purchased by cocoa farmers at the given market price. The right side is cocoa farm household food consumption, given in Eq (1).

Given the highly non-linear nature of this model, the system is solved numerically (the Lagrangian, first-order conditions, and full system of equations are provided in Appendix A in S1 File).

## Data, sources, and calibration

The model presented in the previous section is calibrated to the Ghanaian cocoa market to simulate the effect of removing child labor in cocoa production. This calibration process utilized data from three micro-level sources: (i) the Ghana Living Standards Survey conducted in 2012/13 [37], (ii) Ghana Cocoa Farmers Survey conducted in 2005/06 [36], and (iii) the Tulane Child Labor Survey in Ghana conducted in 2012/13 [12]. All relevant data are within the manuscript and its supporting information files. As summarized in Table 2, these sources provide data on the following variables: the number of cocoa farming households, the value of production inputs per hectare, the annual household budget structure, time use, and cocoa price. Exchange rate used for monetary conversion is 1.954GHC/1$ retrieved from [38]. All monetary values are in 2012/13 USA dollars ($). In addition, data on annual cocoa production and national food balance sheet are obtained from [39], while the price of non-labor production input was collected from the literature [40] and adjusted by inflation.

**Table 2:** Micro data summaries by data sources.

Variable	Data source		
	Tulane [12]	GLSS [36]	GCFS [35]
Survey year	2012/13	2012/13	2005/06
Sample size for estimates in the table	731	1,134	506
Average household size (count)	5.022	4.644	4.397
Avg. cocoa yield (kg/ha)	230.23	331.333	264.373
Total land (ha)	3.14	3.35	9.003
Cocoa share crop ratio	42.71	13.59	-
**Cocoa production inputs Value ($/ha)**			
Total labor	118.94	67.82	-
Fertilizer	170.18	60.50	717.04
Pesticide	85.99	31.28	6.058
Agro-chemicals	63.48	22.17	69.88
Equipment/others	-	21.672	-
**Cocoa production inputs quantity**			
Land (ha)	2.28	1.93	7.11
Hired labor (man-day /ha)	-	-	52.296
Household labor (man-day/ha)	26.82	-	70.04
Fertilizer (kg/ha)	-	-	206.18
Pesticide (L/ha)	-	-	1.83
Fungicide (kg/ha)	-	-	0.12
**Consumption parameters**			
Total expenditure ($/household/year)	-	4,585.37	-
Food expenditure($/household/year)	-	2,442.68	-
Education expenditure ($/household/year)	-	380.65	-
Consumption from own production (%)	-	37.85	-
**Household time use distribution (%)**			
Child education	-	-	-
Adult education	-	-	-
Child farm work	0.49	0.88	-
Adult farm work	4.94	8.54	-
Child cocoa work	0.27	-	-
Adult cocoa work	3.27	-	-
Child non-farm work	0.98	0.22	-
Adult non-farm work	2.80	3.38	-
Child housekeeping	1.40	1.84	-
Adult housekeeping	2.65	4.40	-
Child leisure/sleep	46.27	38.72	-
Adult leisure/sleep	35.35	45.39	-

As discussed in detail below, the parameters and exogenous variables in the model are calibrated to match the prices and output levels during the period 2012–2013. These parameters and exogenous variables include prices for inputs to production (*w*, $P_{x_{j}}$, and *r*) and consumption goods (*P*_*F*_, *P*_*M*_, and *P*_*E*_); output elasticities of inputs to production, labor shares, and productivity parameters for cocoa and household staple food production (*β*,*δ*,*ψ*, and *z*); CES parameter for cocoa labor aggregation (*ρ*); subsistence consumption (*d*); consumption shares (*α*); total available time (*t*); non-cocoa income parameters (*T*); and demand function parameters for Ghanaian cocoa (*η* and *D*_*c*_). Table 3 presents the values of these calibrated parameters and exogenous variables.

**Table 3:** Calibrated parameters.

**Utility function and budget parameters**		**Cocoa production**	
Food budget share (*α*_*F*_)	0.165	Productivity (*Z*_*C*_)	59.272
Non-food budget share (*α*_*M*_)	0.087	Labor input share (*β*_*L*_)	0.488
Adult leisure budget share (*α*_*a*_)	0.128	Fertilizer input share (*β*_*1*_)	0.072
Children leisure budget share (*α*_*e*_)	0.497	Pesticide input share (*β*_*2*_)	0.036
Child education budget share (*α*_*E*_)	0.121	Chemicals input share (*β*_*3*_)	0.026
Education scaler (*κ*)	80.429	Composite input share (*β*_*4*_)	0.038
Subsistence consumption (*d*)	422.008	Hired labor share in total labor (*ψ*_*hc*_)	0.469
Adult total time (*t*_*a*_)^a^	83.586	Adult labor share in total labor (*ψ*_*ac*_)	0.479
Children total time (*t*_*e*_)^a^	114.975	Child labor share in total labor (*ψ*_*ec*_)	0.051
Government Transfer (*T*)^a^	8620.52	labor shares (*ρ*)	−0.500
**Price**		**Food production**	
Food (*P*_*F*_)	2.816	Productivity (*Z*_*F*_)	692.889
Non-food (*P*_*M*_)	2.031	Labor share (*δ*_*L*_)	0.362
Labor (*w*)	0.115	Land share (*δ*_*A*_)	0.063
Education (*P*_*E*_)	38.263	Adult labor share in total labor (*ψ*_*af*_)	0.844
Fertilizer (*P*_*1*_)	0.824	Child labor share in total labor (*ψ*_*ae*_)	0.155
Pesticide (*P*_*2*_)	13.584	**Cocoa market clearing parameters**	
Chemicals (*P*_*3*_)	2.911	Demand elasticity (*ή*)	−0.900
Composite input (*P*_*4*_)	1.000	Demand (*D*_*c*_)	1.802
Land (*r*)	256.63		

^a^ Indicates scaled parameters by 1,000,000

The price of labor ($\bar{w}$) and non-labor inputs (${\bar{P}}_{x_{j}}$) are taken as their respective averages based on the [12] dataset and [40] adjusted by inflation. In Ghana, sharecropping contracts generally are made between farmers and landowners. The two predominant sharecropping contracts in Ghana are *abunu* (division into two shares where the farmer receives 1/2) and *abusa* (division into three shares where the farmer receives 2/3 or 1/3 depending on whether the landowner provides support for inputs). Thus, the rental rate for land ($\bar{r}$) was taken as the average monetary value of cocoa production given to landowners divided by the average cocoa farm size based on [36]. For the consumption goods, the price of food (${\bar{P}}_{F}$), the non-food good (${\bar{P}}_{M}$), and education (${\bar{P}}_{E}$) are calibrated as their average per capita daily values ($/member/day) based on the [37] dataset.

Production parameters for cocoa, *β*_*L*_ and *β*_*j*_, are calibrated as the respective average share of the value of the input to the total value of cocoa production per hectare based on the [12, 30] datasets. Total cocoa production ($\bar{S_{c}}$) per hectare is the average cocoa produced for the period 2012/13 in Ghana retrieved from [38]. Also, the quantities of labor in man-days and non-labor inputs used in production (${\bar{L}}_{c}$, and ${\bar{x}}_{j}$) are calculated based on their respective value per hectare from [12], input prices, and aggregate area planted to cocoa for the period 2012/13 reported by [39]. Given these share parameters, cocoa production, and input data, the cocoa productivity parameter (*Z*_*c*_) in the production function is calibrated as the residual:

$$Z_{c}=\omega^{k}{\bar{S}}_{c}{\left[{\left({\bar{L}}_{c}\right)}^{\beta_{L}}\prod_{j=1}^{4}{\left({\bar{x}}_{j}\right)}^{\beta_{j}}\right]}^{-1}.$$

The value of food consumed ($\bar{F}$) is calculated as the annual average food consumed by cocoa-growing households (based on [36, 37]) divided by the price of food (${\bar{P}}_{F}$). Given $\bar{F}$, the amount of the food produced by the household (${\bar{S}}_{f}$) was calculated as the percentage of food consumption not purchased from the market (Table 2). Because children are less experienced and capable at farming than adults, the CES parameter for cocoa labor aggregation (*ρ*) is taken as −0.5 (or elasticity of substitution of $\sigma =\frac{1}{1-\rho }=0.66$), which ensures that adult and child labor are imperfect substitutes.

The total time available for adults (${\bar{t}}_{a}$) and children (${\bar{t}}_{e}$) measured in man-days is taken as their respective per household values based on [12, 30], multiplied by the number of cocoa-producing households in Ghana. With the average quantity of household labor for both adults (${\bar{L}}_{ac}$) and children (${\bar{L}}_{ec}$) employed in cocoa production collected from [12, 30], the adult (*ψ*_*ac*_) and child (*ψ*_*ec*_) labor share parameters in cocoa production are calibrated as the ratio of their respective values to total labor used in cocoa production. Similarly, the adult (*ψ*_*af*_) and child (*ψ*_*ef*_) labor share parameters in food production are calibrated as the ratio of their respective values (${\bar{L}}_{ac}$ and ${\bar{L}}_{ec}$) to total labor used in food production.

With the total amount of family labor (${\bar{L}}_{ac}+{\bar{L}}_{ec}$) used in the household production of food known, the output elasticities for family labor in food production inputs (*δ*_*L*_) is calibrated as the ratio of the value of family labor to the value of the food produced (${\bar{S}}_{f}\times {\bar{P}}_{F}$). The output elasticity for land (*δ*_*A*_) is calibrated as the ratio of total value of land used in food production to the value of food produced.

The subsistence level of consumption for food ($\bar{d}$) was calculated as the contribution of maize, rice, cassava, and yam to the Recommended Daily Allowance (*RDA*_0_) for calories. According to [39], these four crops provided 42.4% of the total daily caloric intake for the average Ghanaian in 2011. Furthermore, the percent of farming households who harvest cassava, yam, and maize were estimated at 88.0%, 42.7%, and 64.9%, respectively [36, 37]. For the calibration, the study used the *RDA*_0_ value of 2,080 kcal. The value 2,900 kcal is the energy requirements of an adult male between the ages of 19 to 50 presented in [41].

The total value of household consumption expenditures (*EXP*_1_) for food, non-food, and education was taken as their average values from [36, 37]. With the consumption value for food, non-food, and education known, total household consumption is calculated as

$${EXP}_{0}={EXP}_{1}+\bar{w}({\bar{L}}_{a}+{\bar{L}}_{e})+{\bar{E}}_{e}(\bar{w}+{\bar{P}}_{E})-\bar{d}-{\bar{P}}_{F}{\bar{S}}_{f},$$

where ${\bar{L}}_{a}$ and ${\bar{L}}_{e}$ are the average total time households spend at leisure for adults and children collected from [12, 30], and ${\bar{E}}_{e}$ is the total time children spend at education, which was calculated as the product of the percentage of children’s time spent on homework and schooling and times the total available to children (${\bar{t}}_{e}$). The consumption shares parameters were then calibrated as

$$\alpha_{F}=\frac{\bar{F}-\bar{d}-{\bar{P}}_{F}{\bar{S}}_{f}}{{EXP}_{0}};\alpha_{M}=\frac{{EXP}_{1}-\bar{F}-w{\bar{E}}_{e}}{{EXP}_{0}};\alpha_{E}=\frac{{\bar{E}}_{e}(\bar{w}+{\bar{P}}_{E})}{{EXP}_{0}};\alpha_{a}=\frac{\bar{w}{\bar{L}}_{a}}{{EXP}_{0}};\alpha_{e}=\frac{\bar{w}{\bar{L}}_{e}}{{EXP}_{0}}$$

With these shares calibrated, scalar parameter *κ* in the utility function was calibrated as the residual from the first order condition of child education. The non-cocoa income parameter (*T*) was calibrated as the residual income such that the full-income constraint holds with equality.

The demand elasticity for Ghanaian cocoa, *η* = −0.9, is the long-run value reported in [42], which is also presented in Table 2. The scale parameter for the residual Ghanaian cocoa demand is $D_{c}={\bar{S}}_{C}{\left(P_{c}^{\eta }\right)}^{-1}$, where ${\bar{S}}_{c}$ is the total Ghanaian cocoa production and exports given zero domestic consumption. With an inelastic demand elasticity, the derivative of total revenue with respect to cocoa price is greater than zero. Hence, a decrease in *P*_*c*_ due to an increase in cocoa production causes total revenue of cocoa farms to decrease.

Since the elasticity of cocoa supply is not available in the literature, given the relatively small percent of the overall labor market cocoa production accounts for, we assume an elastic elasticity of labor supply of 2. Using this elasticity, wage, and hired worker data, we calibrate the scale parameter as $\omega_{l}=L_{hc}/w^{\gamma_{l}}$. We assume farmers do not produce or purchase enough food to impact the market price. The total food purchased from the market is total food consumption minus food produced by cocoa farmers: *ω*_*f*_ = *F*−*S*_*f*_.

## Simulation analysis

The model is numerically solved (using the “multiroot” function in the “rootSolve” package in R) to analyze the impact of reducing or eliminating child labor on cocoa price and production; food production; farm household consumption; allocation of adult’s time to leisure, cocoa production, and food cultivation; and allocation of children’s time to education, leisure, cocoa production, and food cultivation is quantified. For the baseline scenario, the price premium *σ* is fixed at zero and the child labor restriction ${\bar{L}}_{e}$ is set exactly equal to the allocation of children’s time to cocoa production in the data, which implies that Eq (11) does not affect the equilibrium solution. The baseline model consists of the system of 17 equations in 17 endogenous variables (see Appendix A in S1 File), which, based on the calibration procedure in the previous section, replicates all the values of the endogenous variables given in the data.

Three alternate scenarios for compensating cocoa-producing households for restricting child labor are considered. In the first scenario, the price premium *σ* is endogenously determined such that farm household utility is held constant at the baseline level. In this case, the assumption is that an agent exogenous to the model, such as the government through the COCOBOD or a development agency such as the World Cocoa Foundation, pays for the price premium. In the second scenario, the household is not compensated for the child labor restrictions. Finally, the third scenario allows for the cocoa price premium to be charged to the consumers of Ghanaian cocoa.

During the 20012/13 cocoa growing season, 5,922.3 working hours in agriculture were recorded for 1,562,351 children in [12] over a seven day period. Of this total number, 2.37% of all working hours for children were considered as the worst forms of child labor in cocoa production, as defined by the ILO and in the Literature Review section. Additionally, 9.13% of all working hours for children were considered as both regular work and worst forms of child labor, and 37.49% of all working hours for children were considered as child labor in light work, regular work, and worst forms. Based on these values for each of the three alternate scenarios, three child labor policies are implemented by restricting children’s working hours ${\bar{L}}_{e}$ from the baseline value by (A) 2.37% to eliminate only the worst forms of child labor, (B) 9.13% to eliminate both regular work and worst forms of child labor, and (C) 37.49% to eliminate the light and regular work and the worst forms of child labor.

The results of the three child labor policies for each of the three scenarios are compared to the baseline to determine the impacts on key endogenous variables. Note that, as elaborated in the introduction, children working does not necessarily constitute child labor as defined by the ILO. As such, the main analysis for this paper does not eliminate children’s working hours. Besides, since cocoa production in Ghana is predominantly a household affair, children will at some point in their lives find themselves on the farm taking over family responsibilities from their aging parents.

### Scenario one: Endogenous price premium paid for by COCOBOD

For the first alternate scenario, the government through COCOBOD is responsible for imposing the child labor restriction and paying for the price premium. The model solves for the price premium needed to reduce or eliminate child labor in Ghanaian cocoa production such that household utility is held constant. COCOBOD can thus differentiate their product as child-labor free. To ensure that the child labor restrictions do not negatively impact the welfare of cocoa-growing households, utility is held constant at the baseline level (*U*_*b*_) and the cocoa price premium *σ* is endogenized by including the following constraint in the baseline system of equations:

$$U_{b}={\left(F-d\right)}^{\alpha_{F}}M^{\alpha_{M}}L_{a}^{\alpha_{l_{a}}}L_{e}^{\alpha_{l_{e}}}+{\kappa E}_{e}^{\alpha_{E}}$$ (15)

Therefore, for this scenario, a system of 18 equations in 18 endogenous variables is solved. Table 4 presents the results of reducing child labor.

**Table 4:** Results for reducing or eliminating child labor in cocoa production with constant utility.

Variable	Baseline Values	Child Labor Restriction ${\bar{L}}_{e}$ Increments (Percent Changes)		
		(A)^a^	(B)^a^	(C)^a^
		2.37% Reduction, Worst Forms	9.13% Reduction, regular work and worst forms	37.49% Reduction, Light and regular work and the worst forms
Cocoa price premium (%)	0.000	2.814	11.805	56.271
World cocoa price	3.262	−1.064	−4.133	−12.939
Price paid to farmers	3.262	1.719	7.184	36.050
Net income	3246.966	−0.108	−0.306	−0.067
Production:				
Cocoa	368.436	0.968	3.872	13.282
Food	872.689	−1.283	−5.173	−22.830
Consumption:				
Food	2727.402	−0.061	−0.213	−0.611
Non-Food	1694.965	−0.038	−0.112	−0.100
Time allocation of adults:				
Leisure	74.561	−0.091	−0.381	−1.622
Cocoa production	6.022	2.106	8.649	36.600
Food production	3.003	−1.967	−7.879	−33.120
Time allocation of children:				
Leisure	94.342	0.044	0.164	0.541
Education	19.439	0.041	0.159	0.582
Food production	0.552	−6.260	−22.955	−69.382

^a^ Results for (A), (B), and (C) are reported in percent changes relative to the baseline values.

Implementing the child labor restrictions forces the farm household to deviate from the optimal labor allocation in the baseline, which implies farm revenue must be augmented through positive cocoa price premiums to hold utility at a constant level. Imposing the child labor restriction policies (A), (B), and (C) based on international child labor standards results in cocoa price premiums for Ghanaian farmers of 2.814%, 11.805%, and 56.271%, respectively (in the subsequent text, results for policy (A) is first, followed by the results for policy (B) and then policy (C)).

The premium causes cocoa production to expand (rightward shift in the supply curve), which increases production by 0.968%, 3.872%, and 13.282%, respectively, for policies (A), (B), and (C). Since Ghana only processes a small amount of cocoa into chocolate, the vast majority of production expansion is exported, which reduces the world cocoa price by 1.064%, 4.133%, and 12.939%. Consequently, consumers of Ghanaian cocoa benefit because they can purchase more cocoa at a lower world price. Due to the decline in the world price, the net price received by cocoa farmers (1+*σ*)*P*_*c*_ does not increase by the full amount of the premium: 1.719%, 7.184%, and 36.050%. This result highlights the importance of modeling the world market clearing condition for Ghanaian cocoa.

The increase in cocoa production discussed above is driven by an expansion of total labor and intermediate inputs; however, hired labor increases very minimally for policies (A) and (B) and declines slightly for policy (C) (not reported in Table 4). This, coupled with the restriction on children in cocoa production, implies that the increase in total labor results solely from adult farm labor, which increases by 2.106%, 8.649%, and 36.60%. Farmers prefer to use their own adult labor as opposed to expanding employment of hired labor because, with the wage rate well below the price of cocoa, the opportunity cost of leisure is low. Consequently, it is more cost-effective for farmers to utilize their own time than to hire workers. Note that because adult labor is more productive than child labor (*ψ*_*ac*_>*ψ*_*ec*_), fewer adult-labor hours are needed to replace the restricted child labor hours.

Cocoa farmers engage in food production primarily for subsistence consumption, which is insufficient to meet the household total food needs. Consequently, cocoa farmers also purchase food from the market. However, because cocoa farmers represent a small portion of the overall food market, the food price is not responsive to changes in a cocoa farm household’s food purchases. Consequently, the price of food relative to the net cocoa price declines. This fall in the relative price of food implies farmers can enhance their income by expanding cocoa production and contracting food production. With more money and less home-produced food, farmers purchase more food from the market. As a result, farmers divert labor resources and intermediate inputs from food to cocoa cultivation, leading to a decline in food production of 6.260%, 22.955%, and 69.382%. The decline in total labor in food production comes from a fall in both adult labor (1.967%, 7.879%, and 33.120%) and children working (6.260%, 22.955%, and 69.382%). Interestingly, the child labor restrictions in cocoa production and cocoa price premium not only limit child labor in cocoa production but also reduce the number of hours children spend working in food production.

Even with the cocoa price premium, the child labor restriction results in net income (right side of Eq (6)) declining slightly by 0.108%, 0.306%, and 0.067%. As a result, consumption also declines slightly for both food (0.061%, 0.213%, and 0.611%) and non-food (0.038%, 0.112%, and 0.100%) goods. This decline in consumption goods is less than what it would be without the price premium paid to farmers and is also smaller (in terms of absolute differences) than the decline in production due to food purchases from the market (i.e., the difference between consumption and production).

As cocoa becomes more valuable (rise in cocoa price paid to farmers) to the household, the adults in the household spend less time at leisure (a 0.091%, 0.381%, and 1.622% decline) and food cultivation and more time at cocoa cultivation to help augment production. Therefore, with food and non-food consumption and adult leisure falling, utility is held constant through an expansion of child leisure (0.044%, 0.164%, and 0.541%) and—more importantly—education (0.041%, 0.159%, and 0.582%). This leads to a key result: the restriction (or elimination) of child labor, coupled with a constant utility achieved through cocoa price premiums, not only reduces child labor in cocoa production but also reduces the time children spend in food cultivation while increasing their time spent at leisure and education.

### Scenario two: No compensation to Ghanaian farm household

For the second alternate scenario, the Ghanaian government imposes the child labor restriction without compensating the cocoa farmers through the price premium. As a result, the price premium *σ* is exogenously set at zero, and the constraint given by Eq (15) is excluded from the simulation. The results of the three child labor restriction policies under scenario two are reported in Table 5. Without a cocoa price premium, the child labor restrictions cause farmers to *reduce* cocoa production by 0.290%, 1.172%, and 6.022%, respectively, for policies (A), (B), and (C), as they deviate from the optimal labor allocation under the baseline. This decline in the Ghanaian cocoa supply drives the world cocoa price up by 0.323%, 1.319%, and 7.145%. The increase in the world price does not offset the decline in production, resulting in a decline in net income by 0.382%, 1.534%, and 7.627%, which is more pronounced than in the first alternate scenario with the endogenous price premium.

**Table 5:** Results for reducing or eliminating child labor in cocoa production with variable utility.

Variable	Baseline Values	Child Labor Restriction ${\bar{L}}_{e}$ Scenario (Percent Changes)		
		(A)^a^	(B)^a^	(C)^a^
		2.37% Reduction, Worst Forms	9.13% Reduction, regular work and worst forms	37.49% Reduction, Light and regular work and the worst forms
Cocoa price premium (%)	0.000	0.000	0.000	0.000
World cocoa price	3.262	0.323	1.319	7.145
Price paid to farmers	3.262	0.323	1.319	7.145
Net income	3246.953	−0.382	−1.534	−7.627
Production:				
Cocoa	368.430	−0.290	−1.172	−6.022
Food	872.689	−0.555	−2.230	−11.108
Consumption:				
Food	2727.402	−0.111	−0.447	−2.220
Non-Food	1694.965	−0.125	−0.503	−2.501
Time allocation of adults:				
Leisure	74.561	0.036	0.143	0.722
Cocoa production	6.022	−0.073	−0.301	−1.679
Food production	3.003	−0.736	−2.953	−14.562
Time allocation of children:				
Leisure	94.342	0.029	0.113	0.446
Education	19.439	0.021	0.084	0.369
Food production	0.552	−3.015	−11.557	−45.667

^a^ Results for (A), (B), and (C) are reported in percent changes relative to the baseline values

As in the first alternate scenario, the cocoa price paid to farmers increases, but the increase is a result of a rise in the world price of cocoa and a cocoa price premium. The higher cocoa price implies that the relative price of food falls; however, because the increase in the cocoa price paid to farmers is less pronounced here than in the first alternate scenario, the fall in the relative price of food and, thus, food production (0.555%, 2.230%, and 11.108%) is also less pronounced. With a larger drop in net income compared to the first alternate scenario, the decline in both food (0.111%, 0.447%, and 2.220%) and non-food (0.125%, 0.503%, and 2.501%) consumption is also more pronounced.

The fall in both cocoa and food production results in adults in the household spending more time at leisure (0.036%, 0.143%, and 0.722%) and less time at cocoa (0.073%, 0.301%, 1.679%) and food (0.736%, 2.953%, 14.562%) production. Interestingly, for the children’s time allocation, the directional impacts of the labor restriction remain unchanged. However, because the decline in food production is smaller than in the first alternate scenario, less time is taken away from food production, and the children spend relatively less time at leisure (0.029%, 0.113%, and 0.446%) and education (0.021%, 0.084%, and 0.348%) compared to those in the first scenario.

Therefore, regardless of whether the farm household is compensated for the child labor restriction, the children will allocate less time for cocoa and food production and more time for leisure and education. However, without compensation for the child labor restriction, the overall welfare of the farm household declines, and children spend relatively less time at leisure and education as they do when the farm household is fully compensated. Finally, without compensation, parents have less incentive to abide by the labor restriction, and enforcement is substantially more difficult.

### Scenario three: Consumers of Ghanaian cocoa pay the price premium

For the third alternate scenario, since the majority of Ghanaian cocoa is exported, international consumers of Ghanaian cocoa pay the price premium. In this case, the price premium causes the cocoa demand curve to shift leftward, which results in both the quantity demanded and world price to decline. Simultaneously, the price premium causes production to expand (rightward shift in the cocoa supply), which leads to the quantity supply to rise and the world price to fall. The decline in demand dominates the increase in production, and production falls by the same amount as in the second alternate scenario with no price premium because the premium paid to farmers is exactly offset by the decline in world price (see Appendix B in S1 File for a graphical analysis of this result). As a result, the effect of the child labor restrictions on net farm income (and, thus, utility and all other endogenous variables) is equal to that under the case with no price premium. Consequently, the only impacts are in the international market. Furthermore, because a change in the price premium does not impact utility, the price premium cannot be solved endogenously.

Since *σ* is exogenous, two premiums from policies (A) and (B) in the first alternate scenario (2.814% and 11.805%) are implemented to analyze the effect on the world price. While the price premium shifts the supply to the right, the child labor restriction shifts the supply curve to the left. If the child labor restriction is large enough relative to the subsidy, then the world price could rise (see Appendix B in S1 File for a graphical analysis of this result). Under the three child labor restriction policies, a 2.814% price premium drives the world price down by 2.422% and 1.455% for child labor restriction policies (A) and (B) and up by 4.213% for policy (C). Thus, with a relatively small subsidy, the more restrictive child labor policy (C) causes the cocoa supply to contract enough such that the world price rises. An 11.805% price premium leads to a world price decline of 10.269%, 9.379%, and 4.168%. Therefore, in general, the more stringent the labor restriction, the less responsive the world cocoa price is to an exogenous increase in the premium. In summary, when the consumers pay for the price premium, the world price is distorted while having the exact same impacts on the farmer as with no subsidy.

### Sensitivity analysis

To analyze the robustness of the results, we conduct sensitivity analyses for labor supply elasticity, *γ*_*l*_, and the world demand price elasticity, *η*. The sensitivity analysis of *γ*_*l*_ reveals that the results are not sensitive to changes in this parameter value. For example, increasing *γ*_*l*_ from 2 to 2.5 causes the price premium to rise slightly from 2.814% to 2.819% for labor restriction policy (A) and from 11.805% to 11.825% for policy (B).

The world demand price elasticity is important because it determines the degree to which a change in Ghanaian cocoa supply impacts the world price and net farm income, a key factor in all consumption and time allocation decisions. Therefore, the results are rerun for a marginal change (*η* = 0.9±0.1) in world demand elasticity for Ghanaian cocoa and report the results for key variables (see Appendix C in S1 File for the results of an extended sensitivity analysis on *η*). For this sensitivity analysis, the study focuses only on the results for child labor policy (C), where child labor is reduced by 37.49%.

Under the first alternate scenario where the Cocoa Board pays for the price premium, marginal changes in the world demand elasticity lead to adjustments in the world cocoa price that are offset by the price premium. For example, relative to the results in Table 4, as the elasticity becomes more inelastic (elastic) the world price declines by a larger (smaller) percentage of 14.434% (11.725%), which is offset by the price premium increasing by a larger (smaller) percentage of 59.001% (54.120%). Consequently, the farmer’s income does not change, resulting in the other endogenous variables being unaffected by marginal changes in the elasticity of demand.

However, under the second alternate scenario, the government imposes the child labor restrictions without compensating the farm household through the price premium. As the world demand elasticity becomes more inelastic (elastic) the world price increases by a larger (smaller) percentage of 7.598% (6.744%), relative to the results in Table 5. Consequently, cocoa production declines by a smaller (larger) percentage of 5.690% (6.318%), while food cultivation declines by a larger (smaller) percentage of 11.321% (10.918%). This results in children spending slightly more (less) time at leisure and school. Also, due to the relatively higher world price and smaller decline in cocoa production, farm income decreases by smaller (larger) percentages 7.547% (7.698%). For the third alternate scenario, because of the shift in both the cocoa supply curve and world demand curve for Ghanaian cocoa, the results of this sensitivity analysis are the same as in the second alternate scenario.

## Conclusions

Based on [12], Ghana’s domestic legislation (laws specifying child labor), international agreements (Harkin-Engle Protocol), and consumer support have only led to marginal improvements in the use of *hazardous* forms of child labor in the cocoa sector. The lack of wide-scale abolishment of child labor in the cocoa-growing regions of Ghana may be because the root cause of child labor is poverty. Cocoa production in Ghana is traditionally small plot farming with most producers living at or below the poverty line. As such, very little motivation exists to reduce child labor, which is an asset, without an economic incentive to do so.

The results show that to eliminate the worst forms of child labor would require a 2.814% price premium, to eliminate the regular work and the worst forms of child labor would require an 11.805% premium, and to eliminate light work, regular work, and the worst forms of child labor would require a producer receiving a 56.271% premium. A 56.271% premium seems implausible to pay producers; however a premium of 2.814% and 11.805% appear to be more pragmatic. Previous work [21] concluded that the monetary transfers to low-income countries needed to eradicate child labor immediately significantly exceed the willingness to pay by consumers in more developed countries. While willingness to pay was not part of this study, it does seem feasible that consumers in high-income countries could be willing to pay a marginal premium (2.09%) to eliminate the worst forms of child labor. Further research into whether consumers in cocoa-consuming countries are willing to pay these estimated premiums is needed.

Given the fact that Ghana still has a marketing board that controls all cocoa exports, COCOBOD could be used as a vehicle to pay potential premiums to producers to eliminate child labor. Some of COCOBOD’s stated objectives are “to encourage the production of cocoa; undertake and encourage the processing of cocoa with the aim of *adding value* for export; regulate the internal marketing of cocoa; secure the most favorable arrangements for the purchase, grading and sealing, certification, sale and export of cocoa; and purchase, market and export cocoa and cocoa products produced in Ghana Decree, 1968 NLCD 278, or any other enactment as suitable for export (COCOBOD, 2017).” In fact, the conclusion of [12], the seminal work on the matter, states that considerable costs need to be shared between the governments and private sector stakeholders as well as other international stakeholders to eliminate child labor to the amount set forth by Harkin-Engle. The nexus of private stakeholders and the government is the COCOBOD.

The hope of the policy prescription put forth in this study is to incentivize the elimination of child labor. Ideally, producers would take the premium they received and invest in their children’s education or make investments in their orchards to enhance future productivity. There is the real possibility that producers would simply take the price premium, invest it into more cocoa acreage and use more child labor instead of less. However, given the fact that all cocoa is sold to the COCOBOD, they have the authority, if they choose to use it, to monitor the elimination of child labor. This would be a monumental task for the COOCBOD to monitor its producers to ensure that each producer who received premiums for child-labor free cocoa abided by a set of guidelines. However, unlike the current structure for reducing child labor, this approach provides economic incentives to do so. To effectively address child labor, COCOBOD would need to ensure that the eliminated child labor from the cocoa orchard does not resurface in another farming sector. That is, child labor used in the production of domestically consumed crops is just as detrimental as child labor used in export crops destined for high-income countries. If these incentives only transfer child labor from cocoa to staple crops, then society is made no better off. The key here is that the price (and thus revenue) farmers receive for cocoa rises relative to the price of staple crops. The results show, given the boost in farmer revenue from the estimated cocoa premium, the farm household reduces the total time children work in other non-cocoa farm operation by 5.51% and 20.68%, as well, for the worst forms and regular work. Therefore, this cocoa price premium leaves more time for children to spend at leisure (0.04% and 0.16% increase, respectively) and education (0.03% and 0.13% increase, respectively). These percent increases imply an additional 6.85 and 25.38 days of leisure per household and 1.32 and 5.08 days of education per household. With around 609,000 farm households producing cocoa, these small percent increases could lead to meaningful changes in overall leisure and education.

The results show that, if child labor is eliminated and cocoa farmers are not compensated, farm welfare declines as net income and consumption of food and non-food goods fall. Given the lack of economic incentive, this policy has proven to be ineffective in practice. The results also show that, if the price premium to eliminate child labor is passed to the consumer, the demand for cocoa shrinks, which lowers the cocoa income to farmers, and welfare declines as net income falls. Thus, consumption of food and non-food good falls.

The COCOBOD can make progress in their mission to enhance the competitiveness of Ghana’s cocoa industry by establishing effective policies to produce child-labor free cocoa. These premiums paid to producers need to be viewed as short-term losses to enhance global competitiveness. Otherwise, there could be long-term market share loss as consumers demand cocoa from other production regions. With the introduction of high-yielding hybrid cocoa plants that are disease resistant, cocoa production in Ghana has the potential to increase faster than the 30% increase it had from 2008/14. Potentially, this surge in production can result in a higher demand for labor, some of which could be child labor. As such, the problem could increase in magnitude if steps are not taken to eliminate it soon.

## Supporting information

S1 File Appendices and tables. (DOCX)
