# Supplementary material for: Estimating the economic incentives necessary for eliminating child labor in Ghanaian cocoa production
Source: PLoS One. 2019 Jun 7;14(6):e0217230. doi: 10.1371/journal.pone.0217230 (PMC6555545; doi:10.1371/journal.pone.0217230)
Supplement: S1 File — (DOCX) [file pone.0217230.s001.docx]

# Appendix A

## Lagrangian

The Lagrangian is

$\mathcal{L=}\left( F-d \right)^{\alpha_{F}}M^{\alpha_{M}}L_{a}^{\alpha_{a}}L_{e}^{\alpha_{e}}+{\kappa E}_{e}^{\alpha_{E}}+\lambda\left( I-\left( P_{F}F+P_{M}M+P_{E}E_{e} \right) \right)+\mu\left( \bar{E}_{e}-E_{e} \right)+\emptyset\left( \bar{L}_{ec}-\left( t_{e}-E_{e}-l_{e} \right) \right)$ (A1)

where

$I=\left( 1+\sigma\right)P_{c}Z_{c}\left( L_{c} \right)^{\beta_{L}}\prod_{j=1}^{4} \left( x_{j} \right)^{\beta_{j}}-w\psi_{hc}\left[ L_{c}^{\rho}-\psi_{ac}\left( t_{a}-L_{aF}-l_{a} \right)^{\rho}-\psi_{ec}\left( t_{e}-L_{eF}-E_{e}-l_{e} \right)^{\rho} \right]+P_{F}Z_{f}\left( \psi_{af}L_{af}^{\rho}+\psi_{ef}L_{ef}^{\rho} \right)^{\frac{\delta_{L}}{\rho}}A_{f}^{\delta_{A}}-\sum_{j=1}^{4} \left( P_{j}x_{j} \right)-rA_{f}$(A2)

## First-Order Conditions

The first-order conditions are

Consumption

$\frac{\partial\mathcal{L}}{\partial F}=\alpha_{F}\left( F-d \right)^{\left( \alpha_{F}-1 \right)}M^{\alpha_{M}}L_{a}^{\alpha_{a}}L_{e}^{\alpha_{e}}-\lambda P_{F}=0$ (A3)

$\frac{\partial\mathcal{L}}{\partial M}=\alpha_{M}\left( F-d \right)^{\alpha_{F}}M^{\left( \alpha_{M}-1 \right)}L_{a}^{\alpha_{a}}L_{e}^{\alpha_{e}}-\lambda P_{M}=0$ (A4)

$\frac{\partial\mathcal{L}}{\partial L_{a}}=\alpha_{a}\left( F-d \right)^{\alpha_{F}}M^{\alpha_{M}}L_{a}^{\left( \alpha_{a}-1 \right)}L_{e}^{\alpha_{e}}-\lambda\frac{1}{\rho}w\psi_{hc}^{-\frac{1}{\rho}}\left[ L_{c}^{\rho}-\psi_{ac}\left( t_{a}-L_{aF}-l_{a} \right)^{\rho}-\psi_{ec}\left( t_{e}-L_{eF}-E_{e}-l_{e} \right)^{\rho} \right]^{\frac{1}{\rho}-1}\left( -\rho\psi_{ac}\left( t_{a}-L_{aF}-l_{a} \right)^{\rho-1}\left( -1 \right) \right)=0$ (A5)

$\frac{\partial\mathcal{L}}{\partial L_{e}}=\alpha_{e}\left( F-d \right)^{\alpha_{F}}M^{\alpha_{M}}L_{a}^{\alpha_{a}}L_{e}^{\left( \alpha_{e}-1 \right)}-\lambda\frac{1}{\rho}w\psi_{hc}^{-\frac{1}{\rho}}\left[ L_{c}^{\rho}-\psi_{ac}\left( t_{a}-L_{aF}-l_{a} \right)^{\rho}-\psi_{ec}\left( t_{e}-L_{eF}-E_{e}-l_{e} \right)^{\rho} \right]^{\frac{1}{\rho}-1}\left( -\rho\psi_{ec}\left( t_{e}-L_{eF}-E_{e}-l_{e} \right)^{\rho-1}\left( -1 \right) \right)+\emptyset=0$ (A6)

$\frac{\partial\mathcal{L}}{\partial E_{e}}={\alpha_{E}\kappa E}_{e}^{\left( \alpha_{E}-1 \right)}-\lambda P_{E}-\lambda\frac{1}{\rho}w\psi_{hc}^{-\frac{1}{\rho}}\left[ L_{c}^{\rho}-\psi_{ac}\left( t_{a}-L_{aF}-l_{a} \right)^{\rho}-\psi_{ec}\left( t_{e}-L_{eF}-E_{e}-l_{e} \right)^{\rho} \right]^{\frac{1}{\rho}-1}\left( -\rho\psi_{ec}\left( t_{e}-L_{eF}-E_{e}-l_{e} \right)^{\rho-1}\left( -1 \right) \right)+\emptyset+\mu=0$ (A7)

Cocoa Production

$\frac{\partial\mathcal{L}}{\partial L_{c}}=\lambda\beta_{L}\left( 1+\sigma\right)P_{c}Z_{c}\left( L_{c} \right)^{\left( \beta_{L}-1 \right)}\prod_{j=1}^{4} \left( x_{j} \right)^{\beta_{j}}-\lambda\frac{1}{\rho}w\psi_{hc}^{-\frac{1}{\rho}}\left[ L_{c}^{\rho}-\psi_{ac}\left( t_{a}-L_{aF}-l_{a} \right)^{\rho}-\psi_{ec}\left( t_{e}-L_{eF}-E_{e}-l_{e} \right)^{\rho} \right]^{\frac{1}{\rho}-1}\rho L_{c}^{\rho-1}=0$ (A8)

$\frac{\partial\mathcal{L}}{\partial x_{j}}=\lambda\beta_{j}\left( 1+\sigma\right)P_{c}Z_{c}\left( L_{c} \right)^{\beta_{L}}\prod_{j=1}^{4} \left( x_{j} \right)^{\left( \beta_{j}-1 \right)}=0$ $\forall j$ (A9)

Food Production

$\frac{\partial\mathcal{L}}{\partial L_{af}}=\lambda P_{F}\frac{\delta_{L}}{\rho}Z_{f}\left( \psi_{af}L_{af}^{\rho}+\psi_{ef}L_{ef}^{\rho} \right)^{\left( \frac{\delta_{L}}{\rho}-1 \right)}\rho\psi_{af}L_{af}^{\rho-1}A_{f}^{\delta_{A}}$

$-\lambda\frac{1}{\rho}w\psi_{hc}^{-\frac{1}{\rho}}\left[ L_{c}^{\rho}-\psi_{ac}\left( t_{a}-L_{aF}-l_{a} \right)^{\rho}-\psi_{ec}\left( t_{e}-L_{eF}-E_{e}-l_{e} \right)^{\rho} \right]^{\frac{1}{\rho}-1}\left( -\rho\psi_{ac}\left( t_{a}-L_{aF}-l_{a} \right)^{\rho-1} \right)\left( -1 \right)=0$ (A10)

$\frac{\partial\mathcal{L}}{\partial L_{ef}}=\lambda P_{F}\frac{\delta_{L}}{\rho}Z_{f}\left( \psi_{af}L_{af}^{\rho}+\psi_{ef}L_{ef}^{\rho} \right)^{\left( \frac{\delta_{L}}{\rho}-1 \right)}\rho\psi_{ef}L_{ef}^{\rho-1}A_{f}^{\delta_{A}}$

$-\lambda\frac{1}{\rho}w\psi_{hc}^{-\frac{1}{\rho}}\left[ L_{c}^{\rho}-\psi_{ac}\left( t_{a}-L_{aF}-l_{a} \right)^{\rho}-\psi_{ec}\left( t_{e}-L_{eF}-E_{e}-l_{e} \right)^{\rho} \right]^{\frac{1}{\rho}-1}\left( -\rho\psi_{ec}\left( t_{e}-L_{eF}-E_{e}-l_{e} \right)^{\rho-1} \right)\left( -1 \right)=0$ (A11)

$\frac{\partial\mathcal{L}}{\partial A_{f}}=\lambda\delta_{A}P_{F}Z_{f}\left( \psi_{af}L_{af}^{\rho}+\psi_{ef}L_{ef}^{\rho} \right)^{\frac{\delta_{L}}{\rho}}A_{f}^{\left( \delta_{A}-1 \right)}-\lambda r=0$ (A12)

Constraints

$\frac{\partial\mathcal{L}}{\partial\lambda}=I-\left( P_{F}F+P_{M}M+P_{E}E_{e} \right)=0$ (A13)

$\frac{\partial\mathcal{L}}{\partial\mu}=\bar{L}_{ec}-\left( t_{e}-E_{e}-l_{e} \right)=0$ (A14)

$\frac{\partial\mathcal{L}}{\partial\emptyset}=\bar{E}_{e}-E_{e}=0$ (A15)

## System of Equations for Baseline Analysis

Below is a system of 16 variables ($F,M,L_{a},L_{e},E_{e},L_{c},x_{j},L_{aF},L_{eF},A_{f},\lambda,\mu,\emptyset, and P_{c}$) in 16 equations

Consumption

$\alpha_{F}\left( F-d \right)^{\left( \alpha_{F}-1 \right)}M^{\alpha_{M}}L_{a}^{\alpha_{a}}L_{e}^{\alpha_{e}}-\lambda P_{F}=0$ (A16)

$\alpha_{M}\left( F-d \right)^{\alpha_{F}}M^{\left( \alpha_{M}-1 \right)}L_{a}^{\alpha_{a}}L_{e}^{\alpha_{e}}-\lambda P_{M}=0$ (A17)

$\alpha_{a}\left( F-d \right)^{\alpha_{F}}M^{\alpha_{M}}L_{a}^{\left( \alpha_{a}-1 \right)}L_{e}^{\alpha_{e}}-\lambda\frac{1}{\rho}w\psi_{hc}^{-\frac{1}{\rho}}\left[ L_{c}^{\rho}-\psi_{ac}\left( t_{a}-L_{aF}-l_{a} \right)^{\rho}-\psi_{ec}\left( t_{e}-L_{eF}-E_{e}-l_{e} \right)^{\rho} \right]^{\frac{1}{\rho}-1}\left( -\rho\psi_{ac}\left( t_{a}-L_{aF}-l_{a} \right)^{\rho-1}\left( -1 \right) \right)=0$ (A18)

$\alpha_{e}\left( F-d \right)^{\alpha_{F}}M^{\alpha_{M}}L_{a}^{\alpha_{a}}L_{e}^{\left( \alpha_{e}-1 \right)}-\lambda\frac{1}{\rho}w\psi_{hc}^{-\frac{1}{\rho}}\left[ L_{c}^{\rho}-\psi_{ac}\left( t_{a}-L_{aF}-l_{a} \right)^{\rho}-\psi_{ec}\left( t_{e}-L_{eF}-E_{e}-l_{e} \right)^{\rho} \right]^{\frac{1}{\rho}-1}\left( -\rho\psi_{ec}\left( t_{e}-L_{eF}-E_{e}-l_{e} \right)^{\rho-1}\left( -1 \right) \right)+\emptyset=0$ (A19)

${\alpha_{E}\kappa E}_{e}^{\left( \alpha_{E}-1 \right)}-\lambda P_{E}-\lambda\frac{1}{\rho}w\psi_{hc}^{-\frac{1}{\rho}}\left[ L_{c}^{\rho}-\psi_{ac}\left( t_{a}-L_{aF}-l_{a} \right)^{\rho}-\psi_{ec}\left( t_{e}-L_{eF}-E_{e}-l_{e} \right)^{\rho} \right]^{\frac{1}{\rho}-1}\left( -\rho\psi_{ec}\left( t_{e}-L_{eF}-E_{e}-l_{e} \right)^{\rho-1}\left( -1 \right) \right)+\emptyset+\mu=0$ (A20)

Cocoa Production

$\lambda\beta_{L}\left( 1+\sigma\right)P_{c}Z_{c}\left( L_{c} \right)^{\left( \beta_{L}-1 \right)}\prod_{j=1}^{4} \left( x_{j} \right)^{\beta_{j}}-\lambda\frac{1}{\rho}w\psi_{hc}^{-\frac{1}{\rho}}\left[ L_{c}^{\rho}-\psi_{ac}\left( t_{a}-L_{aF}-l_{a} \right)^{\rho}-\psi_{ec}\left( t_{e}-L_{eF}-E_{e}-l_{e} \right)^{\rho} \right]^{\frac{1}{\rho}-1}\rho L_{c}^{\rho-1}=0$ (A21)

$\lambda\beta_{j}\left( 1+\sigma\right)P_{c}Z_{c}\left( L_{c} \right)^{\beta_{L}}\prod_{j=1}^{4} \left( x_{j} \right)^{\left( \beta_{j}-1 \right)}=0$ $\forall j$ (A22)

Food Production

$\lambda P_{F}\frac{\delta_{L}}{\rho}Z_{f}\left( \psi_{af}L_{af}^{\rho}+\psi_{ef}L_{ef}^{\rho} \right)^{\left( \frac{\delta_{L}}{\rho}-1 \right)}\rho\psi_{af}L_{af}^{\rho-1}A_{f}^{\delta_{A}}$

$-\lambda\frac{1}{\rho}w\psi_{hc}^{-\frac{1}{\rho}}\left[ L_{c}^{\rho}-\psi_{ac}\left( t_{a}-L_{aF}-l_{a} \right)^{\rho}-\psi_{ec}\left( t_{e}-L_{eF}-E_{e}-l_{e} \right)^{\rho} \right]^{\frac{1}{\rho}-1}\left( -\rho\psi_{ac}\left( t_{a}-L_{aF}-l_{a} \right)^{\rho-1} \right)\left( -1 \right)=0$ (A23)

$\lambda P_{F}\frac{\delta_{L}}{\rho}Z_{f}\left( \psi_{af}L_{af}^{\rho}+\psi_{ef}L_{ef}^{\rho} \right)^{\left( \frac{\delta_{L}}{\rho}-1 \right)}\rho\psi_{ef}L_{ef}^{\rho-1}A_{f}^{\delta_{A}}$

$-\lambda\frac{1}{\rho}w\psi_{hc}^{-\frac{1}{\rho}}\left[ L_{c}^{\rho}-\psi_{ac}\left( t_{a}-L_{aF}-l_{a} \right)^{\rho}-\psi_{ec}\left( t_{e}-L_{eF}-E_{e}-l_{e} \right)^{\rho} \right]^{\frac{1}{\rho}-1}\left( -\rho\psi_{ec}\left( t_{e}-L_{eF}-E_{e}-l_{e} \right)^{\rho-1} \right)\left( -1 \right)=0$ (A24)

$\lambda\delta_{A}P_{F}Z_{f}\left( \psi_{af}L_{af}^{\rho}+\psi_{ef}L_{ef}^{\rho} \right)^{\frac{\delta_{L}}{\rho}}A_{f}^{\left( \delta_{A}-1 \right)}-\lambda r=0$ (A25)

Constraints

$$\left[ \left( 1+\sigma\right)P_{c}Z_{c}\left( L_{c} \right)^{\beta_{L}}\prod_{j=1}^{4} \left( x_{j} \right)^{\beta_{j}}-w\psi_{hc}\left[ L_{c}^{\rho}-\psi_{ac}\left( t_{a}-L_{aF}-l_{a} \right)^{\rho}-\psi_{ec}\left( t_{e}-L_{eF}-E_{e}-l_{e} \right)^{\rho} \right]+P_{F}Z_{f}\left( \psi_{af}L_{af}^{\rho}+\psi_{ef}L_{ef}^{\rho} \right)^{\frac{\delta_{L}}{\rho}}A_{f}^{\delta_{A}}-\sum_{j=1}^{4} \left( P_{j}x_{j} \right)-rA_{f} \right]$$

$-\left[ P_{F}F+P_{M}M+P_{E}E_{e} \right]=0$ (A26)

$\bar{L}_{ec}-\left( t_{e}-E_{e}-l_{e} \right)=0$ (A27)

$\bar{E}_{e}-E_{e}=0$ (A28)

The cocoa market clearing conditions is

$\left[ P_{c} \right] Z_{c}\left( L_{c} \right)^{\beta_{L}}\prod_{j=1}^{4} \left( x_{j} \right)^{\beta_{j}}=D_{c}P_{c}^{\eta_{P}}$ (A29)

# Appendix B

Graphical analysis for Scenario Three: Consumers of Ghanaian Cocoa Pay the Price Premium

In this scenario, the consumers of Ghanaian cocoa pay the price premium. As seen in the graph below, the price premium causes the international cocoa demand curve to shift leftward from D to D’, which results in both the quantity demanded and world price to decline (indicated by the movement from equilibrium point A to point B given by the intersection of D’ and S). Simultaneously, the price premium causes production to expands (rightward shift in the cocoa supply from S to S’), which causes the quantity supply to rise and the world price to fall (indicated by the movement from equilibrium point B to point C given by the intersection of D’ and S’). The decline in demand dominates the increase in production and production falls from Q to Q’ and the world price falls from P to P’.

C

B

A

Q

Q’

P’

P

D’

D

S

S’

While the subsidy shifts the supply to the right, child-labor restriction shifts the supply curve to the left. If the child-labor restriction is large enough relative to the subsidy, the world price could rise as see by the movement from equilibrium point E given by the intersection of S” and D’.

S’’

E

A

C

B

Q

Q’

P’

P

D’

D

S

S’

# Appendix C

| **Table A. Sensitivity Results for Reducing or Eliminating Child Labor in Cocoa Production with Constant Utility, Elastic demand** ($\eta=1.4$) | | | | |
| --- | --- | --- | --- | --- |
| Variable | Baseline Values | Child Labor Restriction $\bar{L}_{e}$ Increments (Percent Changes) | | |
|  |  | (A)^a^ | (B)^a^ | (C)^a^ |
|  |  | 2.37% Reduction,  Worst Forms | 9.13% Reduction,  regular work and worst forms | 37.49% Reduction,  Light and regular work and the worst forms |
| Cocoa price premium (%) | 0.000 | 2.422 | 10.132 | 48.726 |
| World cocoa price | 3.262 | -0.686 | -2.677 | -8.523 |
| Price paid to farmers | 3.262 | 1.719 | 7.184 | 36.05 |
| Net income | 3246.966 | -0.108 | -0.306 | -0.067 |
| Production: |  |  |  |  |
| Cocoa | 368.436 | 0.968 | 3.872 | 13.282 |
| Food | 872.689 | -1.283 | -5.173 | -22.83 |
| Consumption: |  |  |  |  |
| Food | 2727.402 | -0.061 | -0.213 | -0.611 |
| Non-Food | 1694.965 | -0.038 | -0.112 | -0.1 |
| Time allocation of adults: |  |  |  |  |
| Leisure | 74.561 | -0.091 | -0.381 | -1.622 |
| Cocoa production | 6.022 | 2.106 | 8.649 | 36.6 |
| Food production | 3.003 | -1.967 | -7.879 | -33.12 |
| Time allocation of children: |  |  |  |  |
| Leisure | 94.342 | 0.044 | 0.164 | 0.541 |
| Education | 19.439 | 0.041 | 0.159 | 0.582 |
| Food production | 0.552 | -6.26 | -22.955 | -69.382 |

To provide a more robust sensitivity analysis on the cocoa demand elasticity, we report the results for ($\eta= 0.9\pm0.5$).

| **Table B. Sensitivity Results for Reducing or Eliminating Child Labor in Cocoa Production with Constant Utility, Inelastic demand** ($\eta=0.4$) | | | | |
| --- | --- | --- | --- | --- |
| Variable | Baseline Values | Child Labor Restriction $\bar{L}_{e}$ Increments (Percent Changes) | | |
|  |  | (A)^a^ | (B)^a^ | (C)^a^ |
|  |  | 2.37% Reduction,  Worst Forms | 9.13% Reduction,  regular work and worst forms | 37.49% Reduction,  Light and regular work and the worst forms |
| Cocoa price premium (%) | 0.000 | 4.198 | 17.862 | 85.824 |
| World cocoa price | 3.262 | -2.379 | -9.059 | -26.785 |
| Price paid to farmers | 3.262 | 1.719 | 7.184 | 36.05 |
| Net income | 3246.966 | -0.108 | -0.306 | -0.067 |
| Production: |  |  |  |  |
| Cocoa | 368.436 | 0.968 | 3.872 | 13.282 |
| Food | 872.689 | -1.283 | -5.173 | -22.83 |
| Consumption: |  |  |  |  |
| Food | 2727.402 | -0.061 | -0.213 | -0.611 |
| Non-Food | 1694.965 | -0.038 | -0.112 | -0.1 |
| Time allocation of adults: |  |  |  |  |
| Leisure | 74.561 | -0.091 | -0.381 | -1.622 |
| Cocoa production | 6.022 | 2.106 | 8.649 | 36.6 |
| Food production | 3.003 | -1.967 | -7.879 | -33.12 |
| Time allocation of children: |  |  |  |  |
| Leisure | 94.342 | 0.044 | 0.164 | 0.541 |
| Education | 19.439 | 0.041 | 0.159 | 0.582 |
| Food production | 0.552 | -6.26 | -22.955 | -69.382 |
